# Pacemaker Relocation for Radiation Against Overlapping Lung Cancer

**DOI:** 10.7759/cureus.49921

**Published:** 2023-12-04

**Authors:** Atsuo Mori, Tohru Kuribayashi, Hirofumi Haida, Koji Funaishi, Hirofumi Kasahara, Yuko Harada, Tatsuji Yoshimoto

**Affiliations:** 1 Cardiovascular Surgery, Kawasaki Municipal Hospital, Kawasaki, JPN; 2 Radiology, Kawasaki Municipal Hospital, Kawasaki, JPN; 3 Cardiovascular Surgery, Hiratsuka City Hospital, Kanagawa, JPN; 4 Internal Medicine, Harada Naika Clinic, Kawasaki, JPN; 5 Cardiology, Kawasaki Municipal Ida Hospital, Kawasaki, JPN

**Keywords:** epicardial lead, pacemaker relocation, radiation therapy, lung cancer, pacemaker

## Abstract

We experienced a patient after pacemaker (PM) implantation who had lung cancer of the left upper lobe that developed just behind the PM. The patient was an 81-year-old man with many complications. Radiation was the only treatment option. The PM had to be moved to another place to avoid direct radiation exposure to it. An epicardial pacing lead was implanted on the right ventricular epicardium, and the new generator was implanted in the abdomen. The patient was treated with a total of 62 Gy of radiotherapy for lung cancer, achieving a temporary shrinkage of the tumor. During the radiotherapy period, the PM functioned well without harmful events. When radiation therapy is needed in cases where the tumor overlaps the PM, relocation surgery using an epicardial pacing lead may be a useful option.

## Introduction

The number of patients with pacemakers (PMs) and implantable cardioverter-defibrillators is increasing, and the opportunities for radiation therapy for these patients are expected to increase. Operational abnormalities such as resetting and oversensing can occur when these devices are irradiated with radiation, and it is necessary to be aware of the risks and take necessary measures during radiation therapy [1,2]. Cardiovascular implantable electronic devices, including PMs and defibrillators, are known to be damaged by irradiation. PMs are said to have a radiation dose tolerance of 2 to 5 Gy. For patients after PM implantation who have malignant tumors in the chest and who require radiotherapy, if the tumor and PM are far apart, irradiation can be performed by minimizing damage to the PM by avoiding the irradiation field from the device in the irradiation plan. However, if lung cancer or other tumor is accidentally located directly behind the PM after implantation, it is impossible to avoid irradiation by itself, no matter how much the irradiation plan might be tactically designed. Here, we report the case of a patient with lung cancer in the left upper lobe that overlapped with a PM, in which the PM was moved to the abdomen using an epicardial lead, and radiation therapy was performed.

## Case presentation

**Figure 1:**
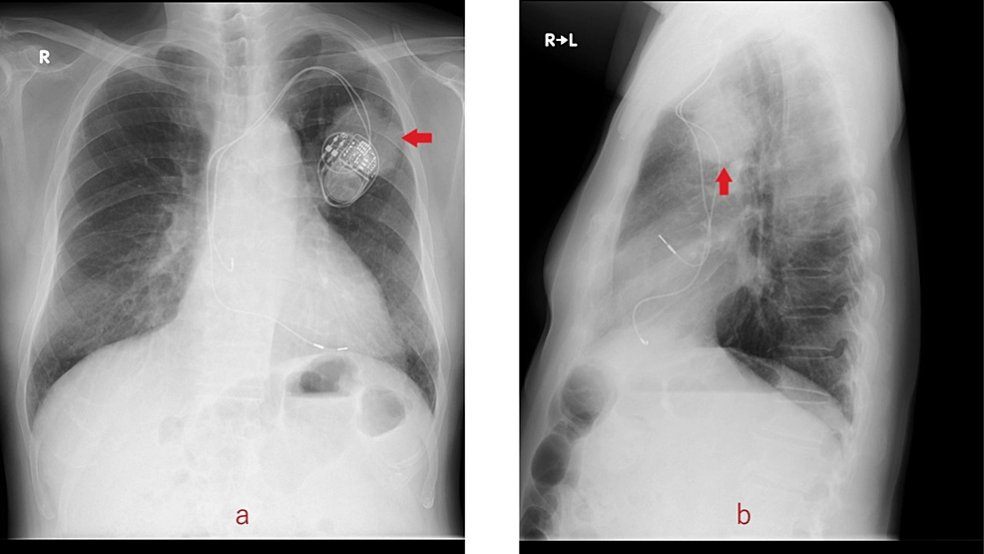
Chest X-ray film. Arrows show a tumor in the left upper lobe of the lung just behind the implanted pacemaker (a: frontal; b: lateral (right to left)).

The patient was an 81-year-old male with a history of type 2 diabetes mellitus, arteriosclerosis obliterans, and chronic renal dysfunction. His laboratory data showed blood urea nitrogen of 67 mg/dL and creatinine of 2.8 mg/dL. He had a history of myocardial infarction and complete atrioventricular block after coronary stenting. He had undergone dual-chamber PM implantation with a transvenous lead in 2015. In 2018, during an outpatient visit, a chest X-ray film showed an enlarging abnormal shadow in the left upper lobe just behind the PM (Figure 1).

**Figure 2:**
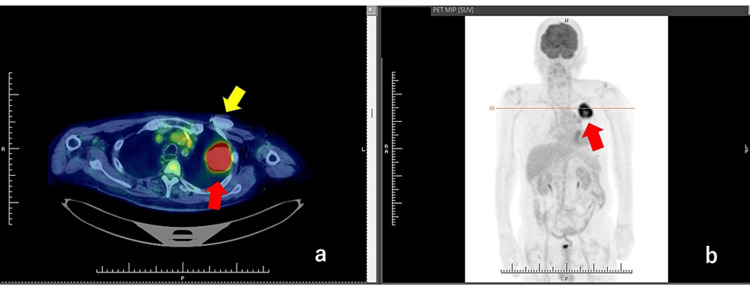
Positron emission tomography. A round mass (red arrow) with a diameter of 57 mm in the left lobe of the lung is shown just behind the pacemaker (yellow arrow).

Positron emission tomography showed an abnormal accumulation, which was strongly suspected to be lung cancer. A mass of about 57 mm in long diameter was found in the left upper lobe, with a high degree of hyperaccumulation (Figure 2).

Lymph node enlargement and hyperaccumulation were observed near the left pulmonary hilum and arch aorta, and lymph node metastasis was suspected. There were no abnormal accumulations that were suggestive of distant metastasis. Sputum pathology detected atypical cells that were suspicious for adenocarcinoma.

Thoracoscopic biopsy was not performed because the surgical risk was estimated to exceed the merit. Because renal dysfunction was also noted, contrast-enhanced CT was not performed. Based on the above, the TNM classification of lung cancer was T3N2M0, and the stage classification was Stage Ⅲb.

Radiation therapy was determined to be the only possible treatment, and the patient agreed to receive it. However, an implanted PM was already in the radiation field for the treatment.

PM transfer to the contralateral side was thought not to be proper because therapeutic radiation from the lateral side to the tumor would damage the PM. Therefore, we decided that PM transfer to the abdominal wall was preferable.

**Figure 3:**
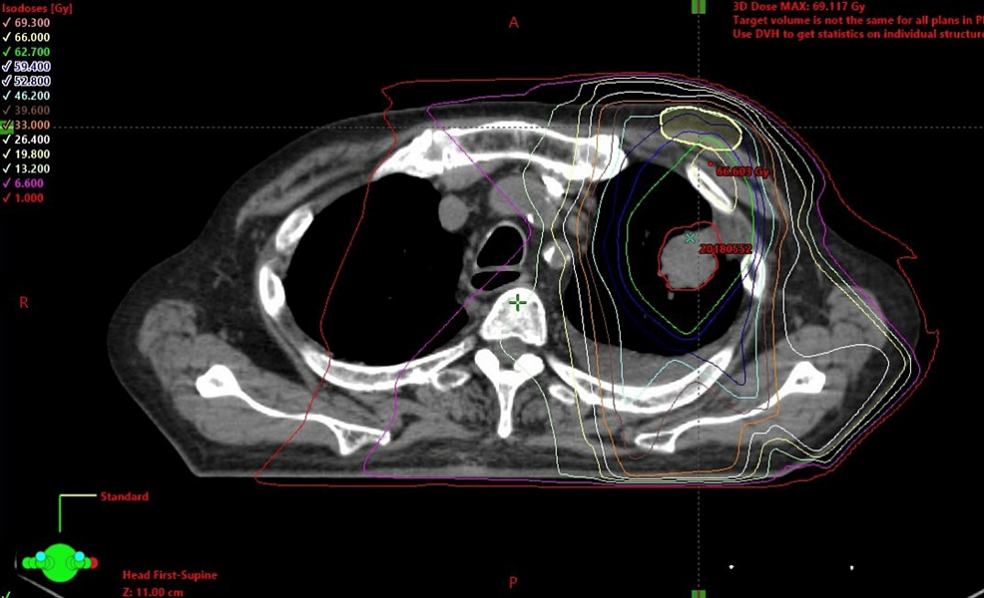
CT before radiation therapy. Estimated irradiation to the pacemaker exceeds 66 Gy.

**Figure 4:**
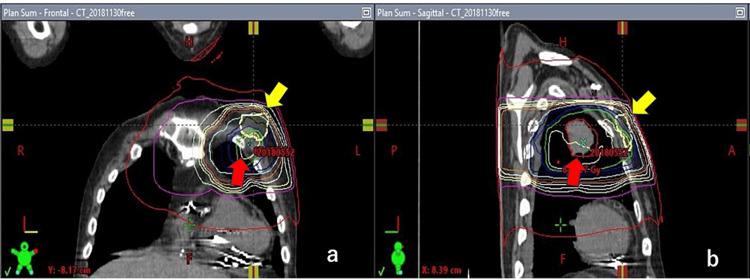
CT before radiation therapy. Exposure dose of the radiation to the pacemaker (yellow arrows) is shown. Red arrows indicate the tumor of the left lung (a: frontal view; b: lateral view).

Radiation dose distribution chart from a treatment planning simulation showed the radiation dose to the PM body would reach 66 Gy, if not moved (Figure 3). It was clear that the device would receive a high dose of radiation in the coronary, sectional, and lateral views (Figure 4). On the other hand, if the PM was moved to the abdominal wall, the radiation dose to the PM was less than 1 Gy, which was less than the permissible standard dose.

**Figure 5:**
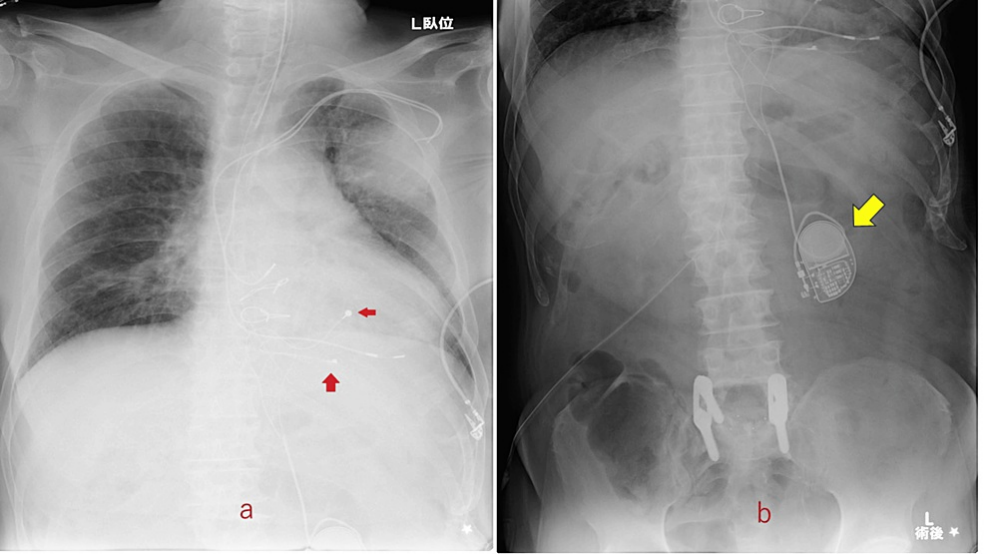
Chest and abdominal X-ray films. Arrows show the electrodes of the new lead attached to the epicardium. The new pacemaker (yellow arrow) implanted in the abdomen.

The operation was performed under general anesthesia and tracheal intubation. The lower half of the sternum was made through a median incision, and the lower half of the pericardium was opened. No artificial heart-lung machine was used, and the surgery was performed under heart beating. One electrode of the epicardial leads was fixed on the anterior surface of the right ventricle and another was fixed on the inferior side. They were not screwing type, but small plate electrode type. The cathode and anode were fixed on the right ventricular epicardium with 4-0 propylene. Pacing threshold, resistance, and sensing sensitivity were measured in situ and were satisfactory. A new PM generator was connected to the epicardial lead and implanted under the rectus abdominis fascia (Figure 5). Then, the old PM generator in the chest wall, which overlapped the lung cancer, was removed. The atrial and ventricular leads were each cut as short as possible, covered with plastic sheath to prevent electronic leakage from the ends, and fixed to the chest wall (Figure 5).

After the operation, the patient suffered from oliguria due to acute renal failure and temporarily required hemodialysis. However, the increase in the amount of diuretic increased self-urine, and dialysis could be withdrawn. At the same time, his cardiac function worsened, requiring oxygen inhalation and the use of diuretics and catecholamine. In addition, diabetes was probably exacerbated by the invasive stress of surgery and anesthesia, resulting in hyperglycemia. We increased the amount of insulin and managed blood glucose within the normal range.

In the next month of the surgery, radiation therapy against the lung tumor was started. The dose of one X-ray irradiation was 2 Gy, and the number of times was 31, for a total of 62 Gy of stereotactic irradiation. A PM check was performed before and after that, but there was no change in the threshold value resistance and sensing value. In addition, there were no events such as a PM reset during this treatment period. There were no complaints from the patient such as fainting, impaired consciousness, dizziness, or convulsions.

**Figure 6:**
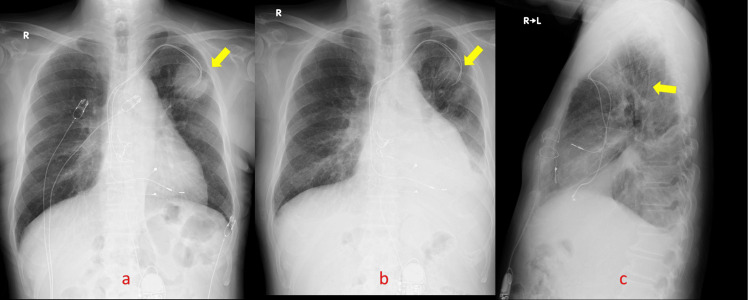
Chest X-ray films. a: The tumor (yellow arrow) three months after the end of radiation therapy (RT). b (frontal view) and c (lateral view): Arrows showing shrinkage of the tumor six months after RT.

For lung cancer, a reduction of the main shadow was observed on chest X-ray films (Figure 6).

However, 18 months after radiation therapy, tumor re-expansion, marked pleural effusion, and bilateral hilar lymph node enlargement were noted. The patient finally expired in 2020.

## Discussion

Radiation therapy is a treatment in which X-rays or neutron beams are delivered to the affected area to damage the DNA of cancer cells. However, it can also damage electronic circuits such as PMs and implantable cardioverter-defibrillators [3,4]. For PMs that are far away from the tumor, it is possible to avoid irradiation by shielding, such as mantle irradiation, or by adjusting the extent and direction of the irradiation field. However, when lung cancer is present just behind the PM, irradiation to the PM cannot be avoided.

According to PM manufacturers, the acceptable dose of radiation exposure for PMs is 2 to 5 Gy, although there is some variation from company to company [3,5]. If the expected exposure to PMs from therapeutic radiation is expected to exceed this amount, it is a matter of debate as to what action is appropriate. The effects of radiation exposure on electronic devices such as PMs can be divided into two categories. One is temporary, in which memory and other settings are altered [3,6]. Another is irreversible damage, which physically destroys the circuitry itself and cannot be normalized by interrogating it. It is also possible that interrogation itself may become impossible due to radiation exposure [7].

The approach to dealing with this problem depends on the distance between the tumor and the PM. First, if the irradiation site and the PM are sufficiently far apart, patients can be treated with normal irradiation. Second, if the tumor and PM are relatively close, it may be possible to reduce the irradiation dose to PM by rearranging the irradiation protocol specifically. However, patients with a 100% pacing rate and no self-replenishment rhythm, or those with a low frequency of self-pulsation and a high pacing threshold, may require backup external pacing. Finally, what should be done in the case of a complete overlapping of tumor and PM, as in this case of lung cancer? If the lung cancer is located directly behind the PM implantation site, it is essential to move the PM to another site [6,7].

The advantages of placing an epicardial lead and transferring the main body to the abdominal wall are that the number of leads passing through the tricuspid valve is not increased and there is no exacerbation of tricuspid regurgitation. In addition, a new lead can be placed even if a mechanical valve has already been placed in the tricuspid valve. The disadvantage of this method is that the chest must be opened for lead placement, and general anesthesia and tracheal intubation may also be required. Compared to conventional local anesthesia surgery, this procedure is more invasive and could be stressful for the patient.

Our patient received radiation therapy without any problems, and throughout the course of 62 Gy of radiation, the PM did not show any abnormalities at any time. The radiation therapy was effective, even if temporarily, and the tumor was reduced in diameter.

## Conclusions

Implantable medical devices, including PMs, have radiation exposure limits. If the tumor targeted for radiation is directly behind the device, there may be situations where it has to be moved to safely deliver radiotherapy. We have reported a case in which the left upper lobe lung cancer overlapped the PM. The PM was moved to the abdomen using an epicardial lead and radiotherapy was performed. The frequency of cases of PM and lung cancer overlapping may increase in the future. We believe that the method reported here may be a useful option for PM relocation.
